# Pulmonary Hypertension

**DOI:** 10.1155/2012/893157

**Published:** 2012-11-13

**Authors:** Ilknur Basyigit, Gulfer Okumus, Serpil Erzurum, Kewal Asosingh, Despina Papakosta

**Affiliations:** ^1^Department of Pulmonary Disease, Faculty of Medicine, Kocaeli University, 41400 Kocaeli, Turkey; ^2^Department of Pulmonary Disease, Faculty of Medicine, Istanbul University, 34452 Istanbul, Turkey; ^3^Department of Pathobiology, Lerner Research Institute (NC22), The Cleveland Clinic, 9500 Euclid Avenue, Cleveland, OH 44195, USA; ^4^Pulmonary Department, Aristotle University of Thessaloniki, G. Papanicolaou Hospital, Exochi, 570 10 Thessaloniki, Greece

Pulmonary hypertension is a hemodynamic and pathophysiological condition which is defined by right heart catheterization as an increase in mean pulmonary artery pressure above 25 mmHg at rest [1]. Several clinical conditions can result in increased pulmonary arterial pressure, therefore detailed evaluation and accurate diagnosis of the underlying disease is crucial for appropriate treatment [2]. The clinical classification of pulmonary hypertension (PH) advanced since its first version proposed by World Health Organization in 1973. The final version of the clinical classification was derived from the Dana Point Meeting in 2008 (Table 1). Pulmonary hypertension due to left heart disease (clinical group 2) is defined as postcapillary (pulmonary capillary wedge pressure ≥ 15 mmHg) while precapillary (pulmonary capillary wedge pressure ≤ 15 mmHg) pulmonary hypertension presents in other groups.

Echocardiography is a widely used screening method in patients with suspected pulmonary hypertension. However, right heart catheterization is required to confirm diagnosis of PH [2]. The evaluation of the patients should also include clarifying specific etiologies and assessment of the degree of functional and hemodynamic impairment. The majority of pulmonary hypertension cases are due to left heart disease and/or lung disease (clinical groups 2 and 3); idiopathic pulmonary arterial hypertension (PAH) remains a diagnosis of exclusion. Hence, diagnostic algorithms are suggested by several guidelines in order to prevent excessive diagnostic testing for a common disease or under-diagnosis of rare conditions [1, 3]. 

Pulmonary hypertension is common and difficult to manage in critical care units (ICU). In the present issue two specific patient populations admitted to ICU are described: pregnant women and newborns. The physiologic changes developing during pregnancy and after labor are poorly tolerated by the pregnant women. Also acute conditions associated with pregnancy such as pulmonary and amniotic fluid embolism may be complicated with severe pulmonary hypertension [4]. No standardized treatment strategies exist for the management of PH in pregnancy, and maternal mortality remains high despite lower death rates in the last decade compared with previous era. 

PH occurring in the newborn may result from a variety of causes, most commonly; it presents immediately after birth and is referred to as persistent pulmonary hypertension of the newborn (PPHN), when pulmonary vascular resistance fails to decrease at birth. The majority of cases are associated with lung parenchymal diseases, such as meconium aspiration syndrome, and respiratory distress syndrome. The improvement in the prognosis and the survival in PPHN over the last decade is attributed to early admission to a tertiary centre, the use of new techniques of mechanical ventilation, extracorporeal membrane oxygenation, and the use of new pulmonary vasodilators [5].

Over the last decade, research in pulmonary vascular disease has revealed genetic mutations in heritable PAH, new methods and imaging techniques for diagnosis of PH, methods to assess right ventricular function and remodeling, and clinical impact of the disease and its prognosis in special conditions such as the pediatric population [6]. 

This special issue aims to identify current limitations as well as future goals to advance the approach to patients with pulmonary vascular disease. The readers will find concise reviews about pulmonary hypertension in newborns and pregnant women, under recognized etiologies of PH and functional impairment of the disease. 

*Ilknur Basyigit* *Ilknur Basyigit* *Gulfer Okumus* *Gulfer Okumus* *Serpil Erzurum* *Serpil Erzurum* *Kewal Asosingh* *Kewal Asosingh* *Despina Papakosta* *Despina Papakosta*

## Figures and Tables

**Table 1:** The clinical classification of pulmonary hypertension [1].

Group 1	Pulmonary arterial hypertension (PAH)
	(i) Idiopathic (IPAH)
	(ii) Heritable (HPAH)
	(a) Bone morphogenetic protein receptor type 2 (BMPR2)
	(b) Activin receptor-like kinase 1 gene (ALK1), endoglin (with or without hereditary hemorrhagic telangiectasia)
	(c) Unknown
	(iii) Drug and toxin induced
	(iv) Associated with (APAH)
	(a) Connective tissue diseases
	(b) Human immunodeficiency virus (HIV) infection
	(c) Portal hypertension
	(d) Congenital heart disease (CHD)
	(e) Schistosomiasis
	(f) Chronic haemolytic anaemia
	(v) Persistent pulmonary hypertension of the newborn (PPHN)

Group 1′	Pulmonary veno-occlusive disease (PVOD) and/or pulmonary capillary haemangiomatosis (PCH)

Group 2	Pulmonary hypertension due to left heart diseases
	(i) Systolic dysfunction
	(ii) Diastolic dysfunction
	(iii) Valvular disease

Group 3	Pulmonary hypertension due to lung diseases and/or hypoxemia
	(i) Chronic obstructive pulmonary disease (COPD)
	(ii) Interstitial lung disease (ILD)
	(iii) Other pulmonary diseases with mixed restrictive and obstructive pattern
	(iv) Sleep-disordered breathing
	(v) Alveolar hypoventilation disorders
	(vi) Chronic exposure to high altitude
	(vii) Developmental abnormalities

Group 4	Chronic thromboembolic pulmonary hypertension (CTEPH)

Group 5	PH with unclear and/or multifactorial mechanisms
	(i) Haematological disorders: myeloproliferative disorders, splenectomy
	(ii) Systemic disorders: sarcoidosis, pulmonary Langerhans cell histiocytosis, lymphangioleiomyomatosis, neurofibromatosis, and vasculitis
	(iii) Metabolic disorders: glycogen storage disease, Gaucher disease, and thyroid disorders
	(iv) Others: tumoral obstruction, fibrosing mediastinitis, and chronic renal failure on dialysis
